# Physicochemical Stability Study of Oral Suspension Containing Ruxolitinib in Children with Steroid-Refractory Acute Graft-Versus-Host Disease

**DOI:** 10.1155/2022/1931118

**Published:** 2022-06-02

**Authors:** Mélanie Hinterlang, Maria Sebti, Camille Cotteret, Fabrice Vidal, Bénédicte Neven, Salvatore Cisternino, Joël Schlatter

**Affiliations:** ^1^Pharmacie, Hôpital Universitaire Necker-Enfants Malades, Assistance Publique-Hôpitaux de Paris (AP-HP), 75015 Paris, France; ^2^Immunohématologie et Rhumatologie, Hôpital Universitaire Necker-Enfants Malades, Assistance Publique-Hôpitaux de Paris (AP-HP), Université de Paris, Institut IMAGINE, 75015 Paris, France; ^3^Inserm, U1144, Université de Paris, Optimisation Thérapeutique en Neuropsychopharmacologie, Paris, France; ^4^Université de Paris, UMRS1144, Paris, France; ^5^Pharmacie, Hôpital Paul Doumer, Assistance Publique-Hôpitaux de Paris (AP-HP), 60140 Labruyère, France

## Abstract

Ruxolitinib, used in children with steroid-refractory acute graft-versus-host (GVH) disease, is currently commercially available only as a tablet adult dosage. For the paediatric population, an oral liquid would be an adapted dosage formulation. The aim of this study was to develop ruxolitinib compounded oral suspensions at 2 mg/mL by using commercial tablets in available aqueous vehicle (Inorpha) and to measure its stability at both room temperature and under refrigeration. Chemical stability of suspensions containing ruxolitinib was evaluated for 60 days based on pH, degradation, and drug content. Physical stability of the drug suspension was evaluated by visual aspect and odour. The remaining ruxolitinib concentration of the suspension was at least 95% of the initial concentration after 60 days at both temperatures. The pH, colour, and odour of the suspensions throughout the study remained unchanged with respect to the initial time point.

## 1. Introduction

Acute graft-versus-host disease (aGVHD) is a critical medical care following allogenic hematopoietic cell transplantation occurring in up to 70% of recipients with a great impact on morbidity and mortality [1, 2]. Actually, the standard initial therapy for aGVHD consists of systemic high-dose corticosteroids at a dose varying from 1 to 2 mg/kg/day that downregulate the production of cytokines and inhibit proliferation of T cells [2, 3]. However, aGVHD remains steroid resistant or refractory in 30–60% of the patients and serious complications causing mortality in 50–70% of cases [4]. Currently, in second-line therapy for steroid-refractory acute graft-versus-host disease (SR-aGVHD), immunosuppressive treatments have been proposed with variable response rates in children [4]. Because Janus kinase (JAK) modulates proinflammatory cytokine signalling, ruxolitinib, an inhibitor of tyrosine kinase JAK1 and JAK2, received approval by the US FDA for the treatment of SR-aGVHD in patients over 12 years old [5]. Its use in patients under 12 years old is still off-label. However, a few preliminary clinical studies evaluate the efficacy and safety of ruxolitinib in this young population suggesting a potential benefit of this treatment in SR-aGVHD [4, 6–8]. The appropriate dose regimen still needs further studies. Ruxolitinib dosing was reported in a panel of 29 patients with a median age at 4.3 y (range, 0.6–14.5 y) that received a median initial dose of ruxolitinib of 12.6 mg/m^2^/day [4]. In Ghobrial et al.' study, a 2-year-old child received daily ruxolitinib 1.25 mg (0.15 mg/kg/dose) per gastric tube [7]. In Khandelwal et al.' study, ruxolitinib was reported to be administered orally at 5 mg twice daily when the child' weight was over 25 kg and 2.5 mg twice daily when the child' weight was below 25 kg [6]. Ruxolitinib is currently only commercially available as a tablet dosage form in the strength of 5 mg, 10 mg, 15 mg, and 20 mg. To achieve the required dose in paediatric patients, the tablets are crushed until a powder is formed. This powder should be then mixed with food or liquid before administration. To limit the risk of an accidental drug exposure among the medical nurses during tablet crushing procedures and loss of drug by preparation of extemporaneous suspension, the availability of a stable pharmaceutical liquid preparation is more convenient [9].

Oral liquid preparation is recommended for children under 6 y and may be used for children of all ages [10, 11]. Since the dose of drug varies according to the volume administered, oral liquid preparation is the choice of preference to reduce the risk of potential dosage errors or inaccuracy and to improve patient and caregiver compliance [9]. No other liquid formulation of ruxolitinib has been found in the literature. The aim of the present work was to formulate a paediatric oral suspension of ruxolitinib and to evaluate its physicochemical stability at 22–25°C and 2–8°C during a 60-day study period.

## 2. Materials and Methods

### 2.1. Chemicals

Commercially available ruxolitinib phosphate tablets were provided by Novartis Pharma (Jakavi® 20 mg tablet, Rueil-Malmaison, France). The Jakavi tablets contained cellulose, magnesium stearate, silica, sodium starch glycolate, povidone K30, hydroxypropylcellulose, and lactose monohydrate. Inorpha® was obtained from Inresa Pharma (Bartenheim, France). According to the manufacturer, Inorpha® oral vehicle is compounded with purified water, glycerol, hydroxyethylcellulose, citric acid, sodium citrate, caramel aroma, sucralose, and potassium sorbate. Inorpha® is a clear and slightly yellowish solution. Its osmolality at 170 mOsm/kg is low, reducing the risk of intestinal aggression by oral administration. The suspended agent Inorpha® contains no excipients with a known effect such as sorbitol or parabens. The buffering effect enables pH stabilization at about 4.7. Moreover, the vehicle contains caramel for the good taste and a bitterness masking agent. Ruxolitinib phosphate reagent quality powder and other chemicals were all of analytical grade (Sigma-Aldrich, Saint Quentin Fallavier, France).

### 2.2. Sample Preparation for One Bottle

A suspension containing ruxolitinib 2 mg/mL was prepared from the commercially available ruxolitinib tablets. Five 20 mg ruxolitinib tablets were crushed in a glass mortar and triturated into a fine powder. The Inorpha® vehicle was added progressively to the powder to obtain a homogenous paste. A volume of 20 mL of Inorpha was added and mixed with a pestle until the homogenization of the mixture was achieved. The mixture was transferred to a volumetric flask and the vehicle was added to obtain a final volume of 50 mL. The latter was then transferred into an amber plastic bottle (pharmaceutical polyethylene terephthalate syrup bottle, Embellia, Charenton-le-Pont, France).

### 2.3. Physicochemical Characterization of Formulation

Amber plastic bottles containing 50 mL of the suspension were stored at 2–8°C (*n* = 3) and at 22–25°C (*n* = 3) in an air-conditioned room for 60 days. Measures were performed in triplicate at day 0, 5, 15, 21, 36, 42, and 60, which included physicochemical testing such as the visual examination of the liquid, pH, and drug concentration after sample homogenization. During the study, each chromatogram was checked at all wavelengths for breakdown products. Preparations were considered stable if physical characteristics were unchanged, and drug concentration remained above 95% of the initial concentration. The colour assessment was studied using a visual examination method. Using identical tubes of colourless and transparent, 1.0 mL of the homogenised sample to be examined was compared with 1.0 mL of freshly made suspension viewing horizontally against a black background. The pH values were measured in triplicate using a digital pH meter (SevenEasy model, Mettler-Toledo, Viroflay, France).

### 2.4. Quantification Method

**Figure 1:**
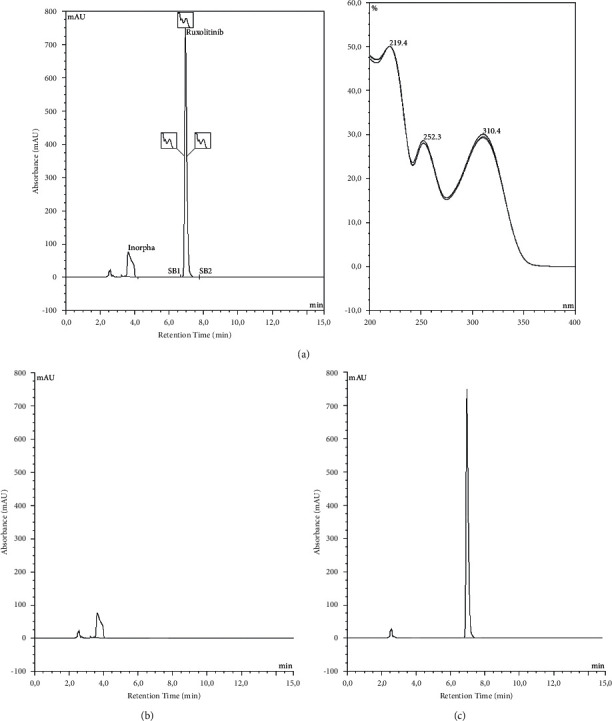
Chromatograms of ruxolitinib in Inorpha® at 200 *μ*g/mL (a), blank with just Inorpha (b), and ruxolitinib alone obtained from an analytical standard at 200 *μ*g/mL (c).

The ruxolitinib content was determined using the stability-indicating high-performance liquid chromatography (HPLC) method previously published by from Douša et al. [12]. Analysis was performed on an integral HPLC system Ultimate 3000 (Thermo-Fisher, Villebon-sur-Yvette, France) equipped with a diode array detector. The chromatographic separation was performed on an octadecyl (C18) silica gel HPLC column (250 × 4.6 mm, 5 *μ*m, Agilent®, Les Ulys, France). The mobile phase consisted of acetonitrile/water with a ratio 40 : 60 (v/v) with isocratic elution set at a flow rate of 1.0 mL/min. The absorbance for ruxolitinib quantification was measured at a wavelength of 295 nm. The typical chromatogram of ruxolitinib in Inorpha® is shown in Figure 1.

In order to calculate the ruxolitinib content in samples, 100 *μ*L of sample equivalent to 200 *μ*g of ruxolitinib was added to 900 *μ*L in a glass volumetric flask, obtaining a final ruxolitinib concentration of 200 *μ*g/mL.

A validation of the adapted methodology was carried out according to the international conference on harmonization guidelines Q2 (R1) including an evaluation of linearity, specificity, sensitivity, accuracy, and precision [13]. The linearity was determined by a least-square linear regression routine by subdividing the calibration curve at five concentration levels from 160 to 240 *μ*g/mL. To compare the absorbance versus nominal concentration of each standard, one-way analysis of variance (ANOVA) was performed. The differences were considered statistically significant when *p* < 0.05. The least-squares linear regression analysis and mathematical determinations were performed by the Prism®, Version 6.01 software (GraphPad Software Inc., San Diego, USA). Limit of detection (LOD) and limit of quantification (LOQ) were determined by the calibration curve method using the following equations:

(1) $$\begin{array}{c} \text{LOD}=\frac{3.3\times \text{SD of intercept}}{\text{Slope of calibration curve}},\\ \text{LOQ}=\frac{10\times \text{SD of intercept}}{\text{Slope of calibration curve}}. \end{array}$$

Accuracy was defined as the percentage of the systematic error between the calculated value and the theoretical value (relative error <10%). Finally, the precision was assessed at intraday and interday precisions. The intraday was determined by measuring control samples of ruxolitinib, injected six times on the same day. Similarly, the intermediate precision (interday) was estimated by injecting control samples on three different days.

In order to assess the specificity, the stability-indicating capability of the chromatographic method was assessed. This was achieved by subjecting ruxolitinib in Inorpha® samples to forced degradation conditions for 6 hours including 1M hydrochloric acid, 1M sodium hydroxide, 3% hydrogen peroxide, and 60°C heat. All samples were stored at 60°C. Therefore, the slopes and intercepts with and without excipients were compared using the ruxolitinib analytical standard to create calibration curves. The peak purity of the drug was performed using the diode array detector comparing peak-controlled spectrum.

## 3. Results and Discussion

### 3.1. Method Validation

**Figure 2:**
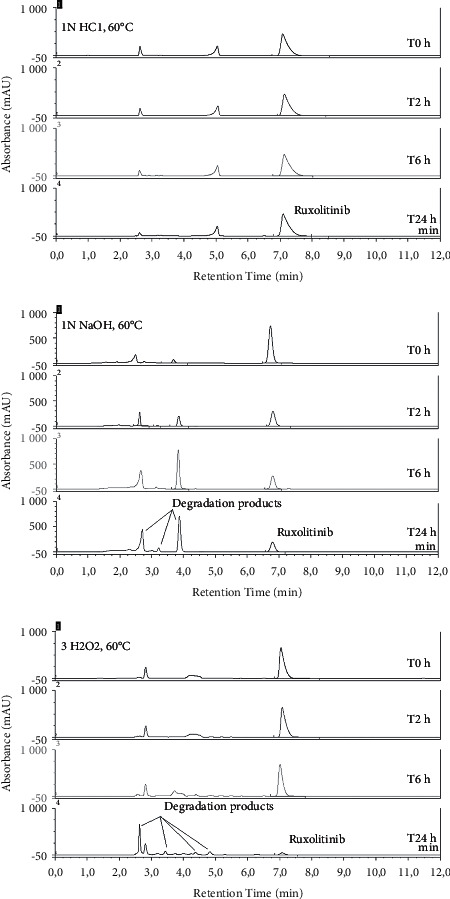
Chromatograms of ruxolitinib 200 *μ*g/mL in 1M HCl, 1M NaOH, and 3% H_2_O_2_ stress condition stored at 60°C during 24 h.

The specificity of the HPLC method was demonstrated for the ruxolitinib formulation. Representative chromatograms of the degradation products are shown in Figure 2.

The ruxolitinib concentrations in these stress conditions over time are given in Table 1.

Indeed, in all the stress conditions experiments, the degradation products detected never interfere with the ruxolitinib peak demonstrating that this HPLC method is stable indicating for reliable ruxolitinib quantification. In acidic stress conditions, the ruxolitinib concentration was maintained over the 24 h period suggesting a better stability of ruxolitinib in acidic milieu (Table 1). At the difference, alkaline stress condition shows a fast decline of ruxolitinib (Table 1). In oxidative stress condition, although ruxolitinib was maintained at 90.5% after 6 h, a fast decline occurred with only 6.0% of ruxolitinib remaining after 24 h. In all these conditions, the degradation products detected never interfere with the ruxolitinib peak demonstrating the stability indications of the HPLC method (Figure 2). The peak purity of the drug compared to that of a pure standard not contained any impurities. However, the deviation from the chromatographic method reported by Dousa et al. justified that better selectivity of ruxolitinib with excipients in Inorpha brings a limitation to the forced degradation tests. It is possible that unseparated and/or undetected degradation products still lurk beneath the ruxolitinib peak, but this can only be resolved using mass spectrometry, which is not yet readily available in most laboratories. The linearity of the method was demonstrated in the interval range of 160–240 *μ*g/mL, with the equation for linear regression *y* = 0.6177 (±0.0006) *x* + 0.0994 (±0.2660), with *r*^2^ >0.999. According to the statistical analysis (ANOVA), the calibration curve was linear (*p* < 0.0001) and slopes and origins were not statistically different. The relative standard deviation (RSD) values were less than 2% for all concentrations tested and confirmed the good precision of the method (Table 2).

The percentage recoveries of ruxolitinib were found to be 99.7%–100.5% with RSD ranging 0.2%–2.1%. The results of the recovery studies demonstrate the accuracy of the proposed method. The calculated values of LOD and LOQ were 1.4 and 4.3 *μ*g/mL, respectively.

### 3.2. Physicochemical Stability

After preparation, the suspension containing ruxolitinib was off-white, opaque with a sweet taste. There were no detectable changes in odour, colour, and taste in any samples over the study period. pH measurements in suspension stored under refrigeration and ambient temperature showed stable pH values except at day 60 with a small decrease of about 0.12 or 0.13 pH units depending on the storage condition (Table 3).

**Figure 3:**
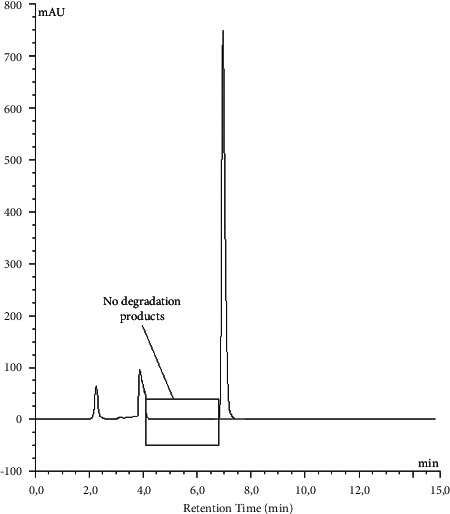
Chromatogram of ruxolitinib in Inorpha® at 200 *μ*g/ml at day 60 and stored at room temperature.

However, this pH decrease had no significant effect on the ruxolitinib concentration in the suspensions. The ruxolitinib concentration remained within 90% of the initial concentration during the 60-day period of storage at two temperatures (Table 3). At different storage times, no additional peaks were noticed at other wavelengths using the diode array detector (Figure 3).

## 4. Discussion

In 2019, the FDA approved ruxolitinib for aGVHD in paediatric patients over 12 y, but no marketed authorization is available in paediatric patients less than 12 y. In this study, we developed an oral suspension containing ruxolitinib in a sweetened vehicle with adequate physicochemical stability allowing liquid preparation in advance. The greater stability of ruxolitinib at acidic pH underlines the importance of developing a pharmaceutical solution in accordance with such chemical property. The oral suspension containing ruxolitinib 2 mg/mL stored in amber plastic bottles at 22–25°C and 2–8°C demonstrated chemical stability up to 60 days. Ruxolitinib retained more than 95% of its initial concentration when admixed with Inorpha® at both storage temperatures. Stability at ambient temperature offers an additional convenience for the storage of the preparation in clinical practice. Microbial testing of the preparations also was not conducted. However, one of the advantages of the commercially available vehicles used in this study is that it is formulated with preservative (potassium sorbate) to prevent microbial growth in extemporaneously prepared products. The manufacturer of Inorpha indicates a stability period of 3 months once the bottle has been opened. Despite this, the absence of a microbiological stability assessment could be a constraint to this study. With regards to the forced degradation, the pH has a high impact on drug stability with a higher stability at acidic pH. Moreover, because ruxolitinib is light sensitive, the drug suspension was formulated into the amber glass container [14]. Taste assessment of the suspension was performed exclusively in our laboratory because this is antineoplastic agent; therefore, the palatability of the suspensions could not be determined by human testing.

## 5. Conclusions

A new oral liquid formulation of ruxolitinib for paediatric use has been developed, derived from the tablet form. This solution was stable from a physicochemical point of view for 60 days at 22–25°C and 2–8°C. The availability of ruxolitinib powder of pharmaceutical grade quality would enable the production of medicines by a simpler preparation process, and this is estimated to result in a purer form and elimination of the tablets' excipients residues. This formulation already contributes to facilitate administration of ruxolitinib for the treatment of paediatric patients who need it.

## Figures and Tables

**Table 1:** Remaining concentration of ruxolitinib phosphate and retention time of degradation products detected.

Stress condition	% remaining			Retention time of degradation peak (min)
	T2 h	T6 h	T24 h	
Acidic (1M HCl, 60°C)	100.9	100.4	100.6	
Alkaline (1M NaOH, 60°C)	41.9	36.2	29.9	2.8; 3.4; 3.9
Oxidative (3% H_2_O_2_, 60°C)	98.2	90.5	6.0	2.8; 3.4; 4.3; 4.8

**Table 2:** Results from the study of precision.

Ruxolitinib concentration (*μ*g/mL)	Intraday precision		Interday precision	
	Mean peak area ± SD (*n* = 6)	RSD (%)	Mean peak area ± SD (*n* = 3)	RSD (%)
170	104.9 ± 0.8	0.8	104.71 ± 0.8	0.8
200	124.0 ± 1.0	0.9	123.62 ± 0.8	0.7
230	142.3 ± 1.0	0.7	142.54 ± 0.9	0.6

**Table 3:** Stability of ruxolitinib phosphate 2 mg/mL oral solution stored at 22–25°C and 2–8°C for 60 days.

Storage	% ruxolitinib concentration remaining (mean ± SD) (*n* = 3)							
	Initial concentration (mg/mL) Day 0	Day 5	Day 15	Day 21	Day 28	Day 36	Day 42	Day 60
22–25°C	2.08 ± 0.01	100.2 ± 0.6	100.9 ± 1.8	100.6 ± 1.1	100.8 ± 0.5	99.5 ± 0.8	97.8 ± 1.5	96.6 ± 0.5
2–8°C	2.07 ± 0.02	100.9 ± 1.9	101.8 ± 1.5	101.2 ± 1.2	99.2 ± 1.4	99.1 ± 1.8	100.6 ± 1.1	99.4 ± 0.6

Storage	pH value (mean ± SD) (*n* = 3)							
	Day 0	Day 5	Day 15	Day 21	Day 28	Day 36	Day 42	Day 60

22–25°C	4.49 ± 0.01	4.45 ± 0.02	4.44 ± 0.01	4.44 ± 0.01	4.40 ± 0.01	4.40 ± 0.02	4.39 ± 0.01	4.36 ± 0.01^*∗*^
2–8°C	4.48 ± 0.01	4.47 ± 0.01	4.47 ± 0.01	4.44 ± 0.02	4.43 ± 0.01	4.42 ± 0.02	4.39 ± 0.01	4.36 ± 0.01^*∗*^

^*∗*^ *P* < 0.0001 (one-way ANOVA).

## Data Availability

The data used to support this study are included within the article.
